# Exogenous C-type natriuretic peptide therapy for impaired skeletal growth in a murine model of glucocorticoid treatment

**DOI:** 10.1038/s41598-019-44975-w

**Published:** 2019-06-12

**Authors:** Yohei Ueda, Akihiro Yasoda, Keisho Hirota, Ichiro Yamauchi, Takafumi Yamashita, Yugo Kanai, Yoriko Sakane, Toshihito Fujii, Nobuya Inagaki

**Affiliations:** 1 Department of Diabetes, Endocrinology and Nutrition, Kyoto University Graduate School of Medicine, 54 Shogoin-Kawahara-cho, Sakyo-ku, 606-8507 Kyoto Japan; 2 Department of Diabetes and Endocrinology, Osaka Red Cross Hospital, 5-30 Fudegasaki-cho, Tennoji-ku, 543-8555 Osaka Japan; 3 Preemptive Medicine and Lifestyle Related Disease Research Center, Kyoto University Hospital, 54 Shogoin-Kawahara-cho, Sakyo-ku, 606-8507 Kyoto Japan; 4 Present Address: Clinical Research Center, National Hospital Organization Kyoto Medical Center, 1-1 Mukaihata-cho, Fukakusa, Fushimi-ku, 612-8555 Kyoto Japan

**Keywords:** Growth disorders, Translational research

## Abstract

Growth retardation is an important side effect of glucocorticoid (GC)-based drugs, which are widely used in various preparations to treat many pediatric diseases. We investigated the therapeutic effect of exogenous CNP-53, a stable molecular form of intrinsic CNP, on a mouse model of GC-induced growth retardation. We found that CNP-53 successfully restored GC-induced growth retardation when both dexamethasone (DEX) and CNP-53 were injected from 4 to 8 weeks old. Notably, CNP-53 was not effective during the first week. From 4 to 5 weeks old, neither CNP-53 in advance of DEX, nor high-dose CNP-53 improved the effect of CNP. Conversely, when CNP-53 was started at 5 weeks old, final body length at 8 weeks old was comparable to that when CNP-53 was started at 4 weeks old. As for the mechanism of resistance to the CNP effect, DEX did not impair the production of cGMP induced by CNP. CNP reduced Erk phosphorylation even under treatment with DEX, while CNP did not changed that of p38 or GSK3β. Collectively, the effect of CNP-53 on GC-induced growth retardation is dependent on age in a mouse model, suggesting adequate and deliberate use of CNP would be effective for GC-induced growth retardation in clinical settings.

## Introduction

Glucocorticoid (GC)-based drugs are widely used to treat many pediatric diseases, such as autoimmune diseases and lymphoproliferative diseases^1–3^. GCs have manifold side effects, and Cushing’s syndrome in childhood mainly results from exogenous GC^4^. As a side effect of GC, GC-induced growth retardation with skeletal impairment is well known and also is well studied^5,6^. GC-induced growth impairment correlates with the dose of GC and becomes apparent when the GC dose exceeds the equivalent of 0.2 mg/kg/day prednisone^7^. Alternate-day treatment can also cause growth impairment and result in reduction of adult height, even if GC therapy has been interrupted^8^. Furthermore, it was recently reported that high-dose inhaled GCs for childhood asthma might be associated with diminished linear growth^9^. As for the mechanisms of GC‐induced growth impairment, there exist many studies: GCs have direct effects on chondrocytes in the growth plate; GCs induce apoptosis, impair differentiation, prevent proliferation of growth plate chondrocytes; and collectively, inhibit endochondral bone growth^10,11^. In addition, GCs are reported to impair the anabolic effects of the growth hormone (GH)/IGF-1 axis on growth plate chondrocytes^12,13^. Accordingly, the effect of GH therapy was reported to be limited when attempting to restore GC-induced growth retardation^14^ — effective therapy for GC-induced growth retardation has not yet been established.

C-type natriuretic peptide (CNP) is the third member of the natriuretic peptide family^15,16^ and is known to be a specific ligand of natriuretic peptide receptor B (NPR-B), which produces cGMP when bound with CNP. CNP stimulates endochondral ossification and promotes linear growth, which was revealed by past experiments using knockout rodents^17–19^ and transgenic mice^20,21^. Furthermore, in humans, some genetic disorders with impaired skeletal growth or skeletal overgrowth phenotype are related to the CNP/NPR-B system. Acromesomelic dysplasia, Maroteaux type, a form of skeletal dysplasia which exhibits short-limbed dwarfism, is caused by biallelic loss-of-function mutations in *NPR2*, which encodes NPR-B^22,23^. Additionally, monoallelic loss-of-function mutations in *NPR2* are reported to exhibit short stature^24–26^. As for the mutation of the gene encoding CNP, a recent study has reported that mutations in *NPPC* cause human autosomal dominant short stature and shortened hands^27^. In contrast, overexpression of CNP caused by a chromosomal translocation exhibits an overgrowth phenotype^28,29^ and monoallelic gain-of-function mutations in *NPR2* also cause a skeletal overgrowth phenotype^30–33^. These findings indicate that CNP/NPR-B signaling plays a major role in endochondral bone growth in humans as well as in rodents. Based on this research, we started performing translational research on the activation of the CNP/NPR-B system to restore skeletal impairment, and previously reported the efficacy of the activation of the CNP/NPR-B system on a mouse model of achondroplasia by using a transgenic approach or intravenous injection of synthetic CNP-22, one molecular form of CNP^20,34^.

We expected that CNP/NPR-B activation would be effective on various skeletal impairments other than achondroplasia. As a prevalent growth retardation, we focused on GC-induced growth retardation. Firstly, we reported that CNP could be a therapeutic agent for GC-induced growth retardation by using transgenic mice that had elevated circulating levels of CNP^35^. However, this past study has a limitation: the CNP transgenic mouse, which produces abundant CNP in the liver under the control of human serum amyloid-P (SAP) component promoter, has elevated CNP levels from its birth^36^ and could not be a rigorous therapeutic model of acquired disease. Therefore, administration experiments of exogenous CNP are necessary to validate and further investigate the optimal effect of CNP for GC-induced growth retardation in clinical settings. In this study, we demonstrated the efficacy of exogenous CNP administration for a mouse model of GC-induced growth retardation, for the first time. Of note, we used CNP-53, the other molecular form of CNP different from CNP-22, as an exogenous CNP preparation, because CNP-53 is the dominant form of endogenous CNP^37^ and resistant to the intrinsic degradation system^38^. As a clinically feasible treatment scheme, we performed daily subcutaneous injection of CNP-53 to GC-treated mice, and altered the start point and dose of CNP-53 to explore the best treatment strategy. In this process, we obtained a mechanistic insight into the effect of CNP on GC-induced growth retardation and further performed some experiments to elucidate this mechanism.

## Results

### The effects of exogenous CNP-53 injection on GC-induced impairment of skeletal growth treated from 4 weeks of age

**Figure 1:**
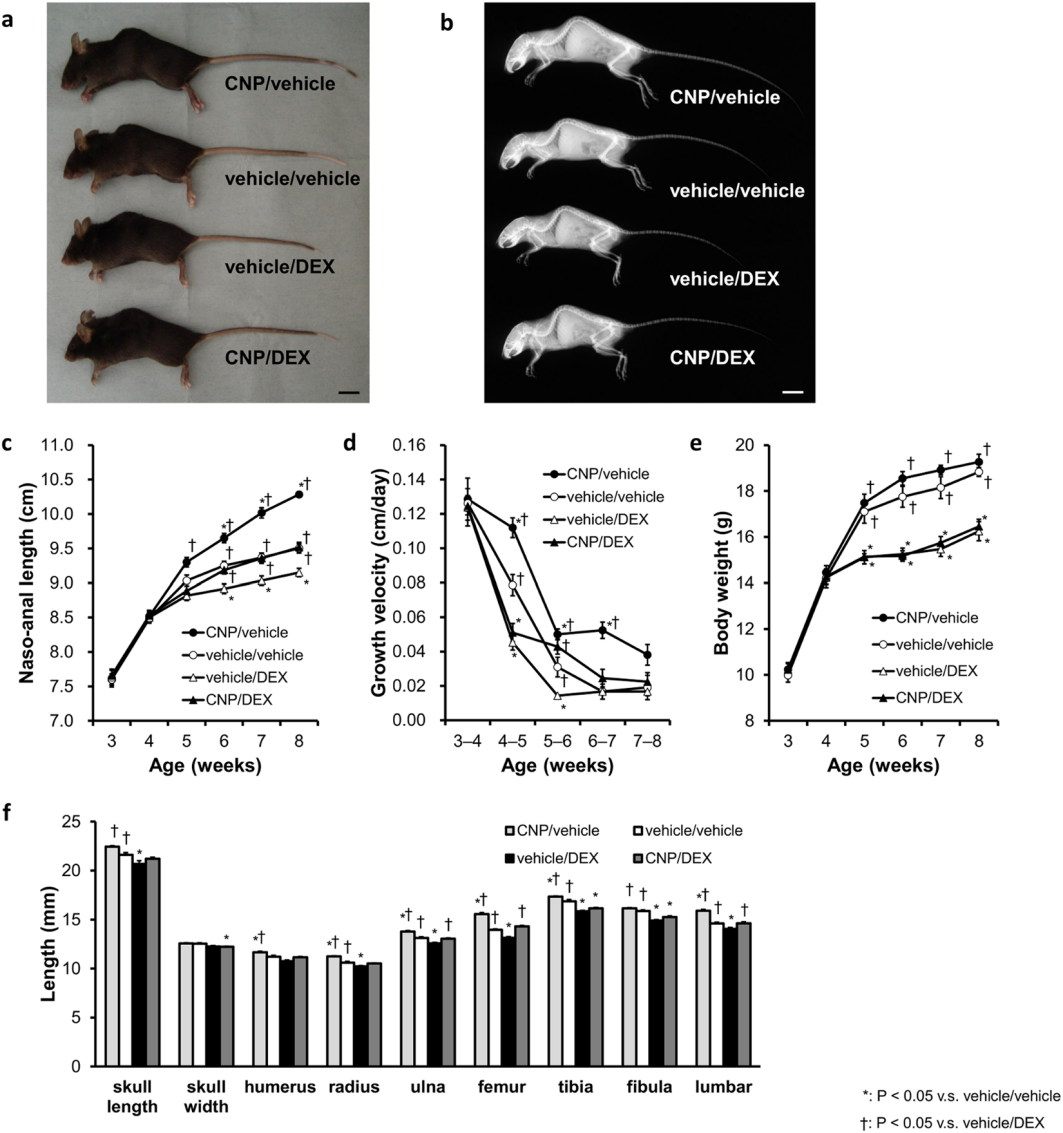
The effect of CNP-53 administration from 4 weeks of age. (**a**) Gross appearances and (**b**) soft X-ray images of CNP/vehicle, vehicle/vehicle, vehicle/DEX, and CNP/DEX mice after the treatment for 4 weeks (8 weeks old). Scale bar in each panel indicates 10 mm. (**c**) Growth curves and (**d**) growth velocities of naso-anal lengths and (**e**) body weights of CNP/vehicle (●), vehicle/vehicle (○), vehicle/DEX (Δ), and CNP/DEX (▲). (**f**) Bone lengths after the treatment for 4 weeks (8 weeks old). CNP/vehicle, n = 7; vehicle/vehicle, n = 6; vehicle/DEX, n = 6; and CNP/DEX, n = 6. (**c**–**f**) *P < 0.05, vs. vehicle/vehicle and ^†^P < 0.05, vs. vehicle/DEX.

We arranged four groups of C57BL/6JJcl mice. The first group was composed of mice treated with CNP-53 at a dose of 0.5 mg/kg/day and saline as a vehicle for dexamethasone (DEX) (CNP/vehicle group). The second was the control group composed of mice treated with water and saline as vehicles for CNP-53 and DEX, respectively (vehicle/vehicle group). The third was composed of mice treated with water as a vehicle for CNP-53 and DEX at a dose of 2 mg/kg/day (vehicle/DEX group). The last was composed of mice treated with CNP and DEX at the same doses, 0.5 and 2.0 mg/kg/day, respectively (CNP/DEX group). We started the administration at 4 weeks of age. The gross appearance and soft X-ray images at the end of the 4-week administration period showed that the vehicle/DEX group exhibited short length due to impaired skeletal growth, which was restored in the CNP/DEX group (Fig. 1a,b). Mice treated with CNP exhibited overgrowth but did not have apparent bone deformity (Fig. 1b).

Growth curves are shown in Fig. 1c–e. There were no significant differences in the naso-anal length between the four groups at the start of this study (4 weeks old). Naso-anal lengths in the vehicle/DEX group became significantly smaller than those of the vehicle/vehicle group. There were no significant differences between the vehicle/vehicle group and the CNP/DEX group (Fig. 1c). However, during the first week from the start of DEX (from 4 to 5 weeks of age), the growth velocity of the CNP/DEX group was not restored compared to that of the vehicle/DEX group (Fig. 1d). After 5 weeks of age, the growth velocity of the CNP/DEX group exceeded that of the vehicle/DEX group and finally the CNP/DEX group became longer than the vehicle/DEX group and comparable to the vehicle/vehicle group (Fig. 1c,d). The body weights of the four groups were not significantly different at the start of the study (Fig. 1e). The body weights of mice treated with DEX became significantly lighter than those not treated with DEX (Fig. 1e). In contrast, CNP treatment did not change the body weight. These results are consistent with our past study using transgenic mice^35^. Next, we measured the length of each bone of the four groups of mice at the end of the experimental period. Skeletal impairments were shown in the vehicle/DEX group and these impairments were restored in the CNP/DEX group. The lengths of the humerus, radius, ulna, femur, and lumbar vertebrae in the CNP/DEX group became comparable to those in the vehicle/vehicle group, whereas those of the tibia and fibula were not fully restored (Fig. 1f). CNP-53 treatment did not widen the skull, although it did lengthen the skull (Fig. 1f).

### Histological examination of the effect of CNP-53 on the growth plate of GC-treated mouse model

**Figure 2:**
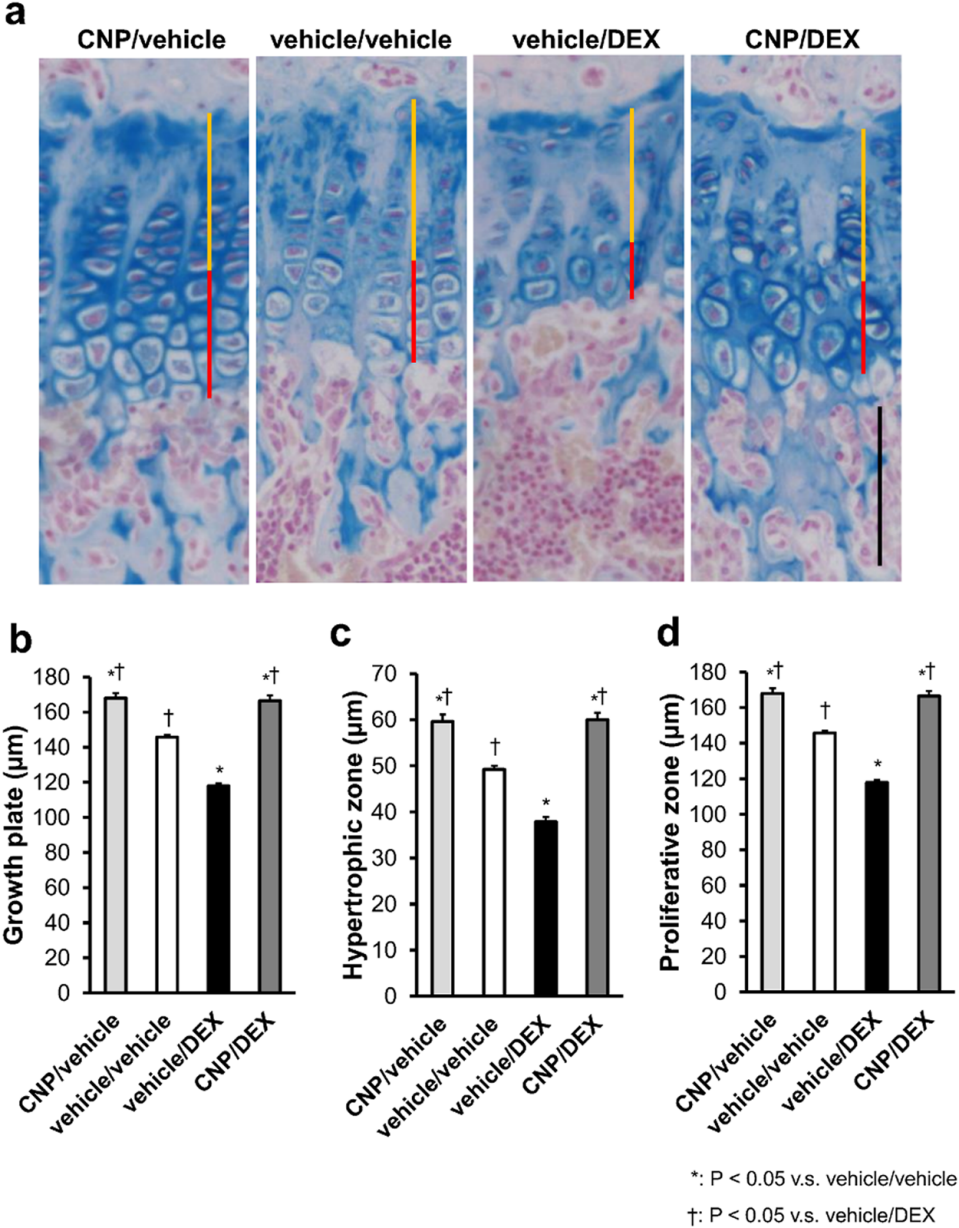
Histological images of growth plates and the width of each chondrocyte layer measured on the pictures. (**a**) The histological images of growth plates of alcian-blue staining. Yellow and red bars indicate the widths of the proliferative and hypertrophic zones, respectively. Black scale bar indicates 100 µm. (**b**–**d**) Graphs of widths of (**b**) whole growth plates, (**c**) hypertrophic zones, and (**d**) proliferative zones measured on histological images of alcian-blue staining. n = 4, each, in the CNP/vehicle, vehicle/vehicle, vehicle/DEX, and CNP/DEX groups. *P < 0.05, vs. vehicle/vehicle and ^†^P < 0.05, vs. vehicle/DEX.

We performed a histological analysis of the tibial growth plates at the end of the experimental period. As shown in the images of alcian-blue staining of growth plates, growth plates of the vehicle/DEX group were impaired and those of the CNP/DEX group were restored (Fig. 2a). The whole growth plates were thinned in the vehicle/DEX group compared with the vehicle/vehicle group and the impairment was restored in the CNP/DEX group (Fig. 2b). Similar results were observed in the proliferative and hypertrophic zones of the growth plates in the four groups (Fig. 2c,d).

### Histological examination of the effect of CNP-53 administered for 3 or 10 days

**Figure 3:**
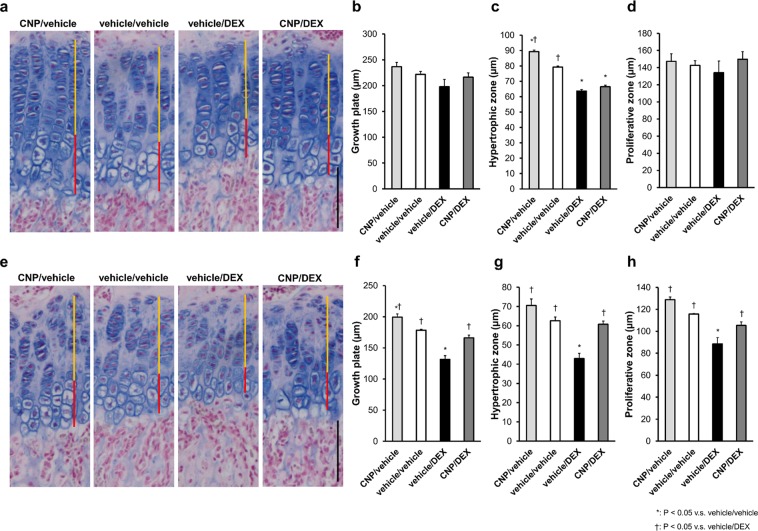
Histological analysis of growth plates of mice treated for 3 and 10 days. (**a**,**e**) Alcian-blue staining of growth plates of mice treated for (**a**) 3 and (**e**) 10 days. Yellow and red bars indicate the widths of the proliferative and hypertrophic zones, respectively. Black scale bar indicates 100 µm. (**b**,**f**) The widths of whole growth plates measured on histological images of alcian-blue staining after treatment for (**b**) 3 and (**f**) 10 days. (**c**,**g**) The widths of hypertrophic zones measured on histological images of alcian-blue staining after treatment for (**c**) 3 and (**g**) 10 days. (**d**,**h**) The widths of proliferative zones measured on histological images of alcian-blue staining after treatment for (**d**) 3 and (**h**) 10 days. (**b**–**d**,**f**–**h**) n = 3, each, in the CNP/vehicle, vehicle/vehicle, vehicle/DEX, and CNP/DEX groups. *P < 0.05, vs. vehicle/vehicle and ^†^P < 0.05, vs. vehicle/DEX.

We focused on the fact that CNP-53 had no effect during the first week after the start of the treatment. To compare the period when CNP had no effect (the first week, at 4–5 weeks old) with that when CNP restored DEX-induced growth retardation (the next week, at 5–6 weeks old), we performed histological analysis in the following two groups: one group treated with CNP-53, DEX, and/or vehicle for 3 days from 4 weeks old and the other group treated with CNP-53, DEX, and/or vehicle for 10 days from 4 weeks old. Whole growth plate widths were not improved in mice treated for 3 days but improved in mice treated for 10 days (Fig. 3a,b,e,f). The proliferative zones and hypertrophic zones of the growth plates of mice treated for 3 days were not widened by CNP-53 when treated with DEX (Fig. 3a,c,d). In contrast, 10 days of treatment with CNP-53 restored DEX-induced impairment of the proliferative zones and hypertrophic zones of growth plates (Fig. 3e,g,h).

### The effects of CNP-53 on GC-induced impaired skeletal growth treated starting one week later

**Figure 4:**
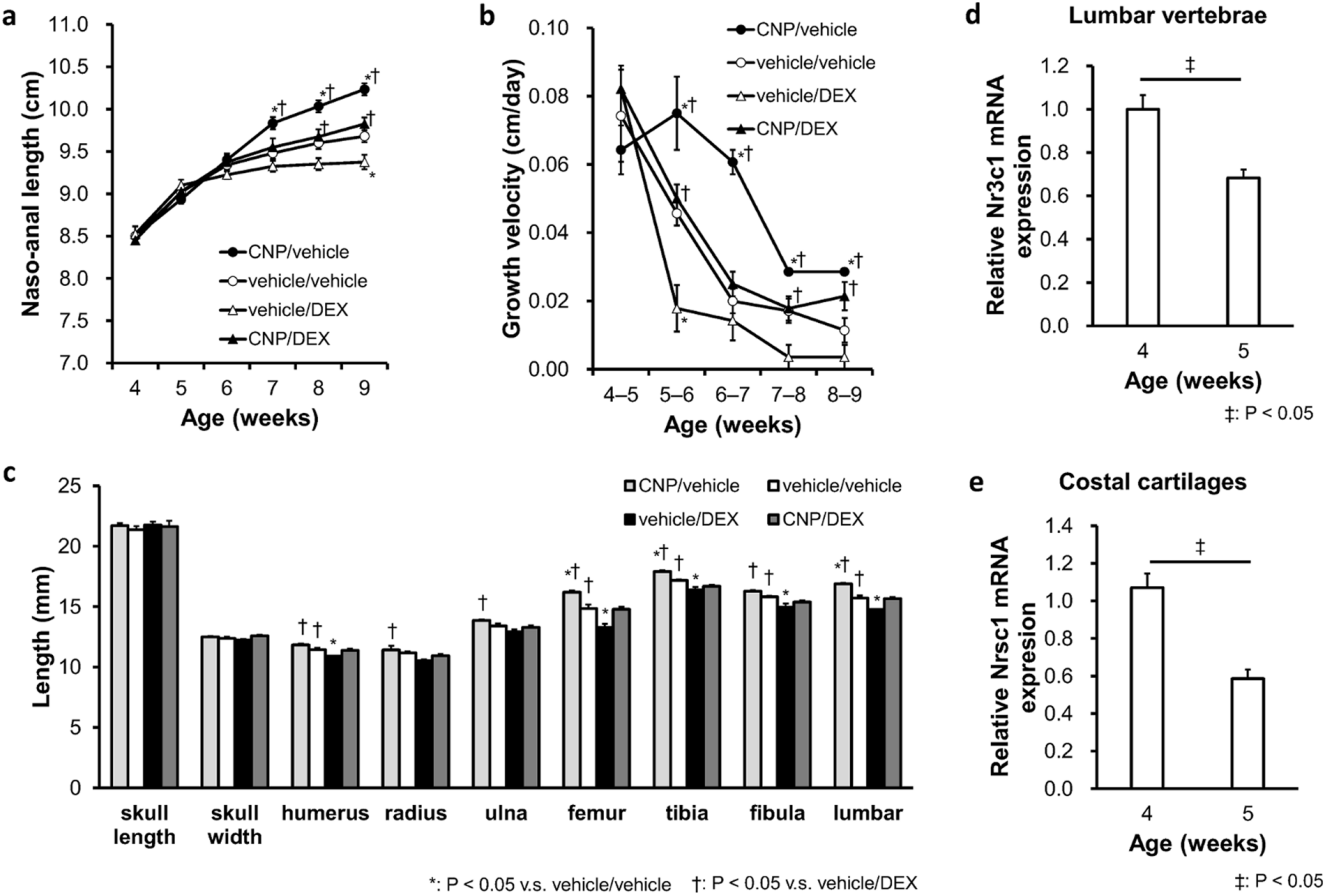
The effect of CNP-53 administration from 5 weeks of age. (**a**) Growth curves and (**b**) growth velocities of naso-anal lengths of CNP/vehicle (●), vehicle/vehicle (○), vehicle/DEX (Δ), and CNP/DEX (▲). (**c**) Bone lengths after treatment for 4 weeks (9 weeks old). (**d**,**e**) The mRNA levels of *Nr3c1* which encodes the murine glucocorticoid receptor normalized by using *Hprt* as the reference gene. (**d**) Lumbar vertebrae and (**e**) costal cartilages. The data are represented as fold-change versus the values for 4-week-old mice. (**a**–**c**) CNP/vehicle, n = 5; vehicle/vehicle, n = 4; vehicle/DEX, n = 4; and CNP/DEX, n = 4, and (**d**–**e**) n = 3, each, in 4-week-old and 5-week-old mice. (**a**–**c**) *P < 0.05, vs. vehicle/vehicle and ^†^P < 0.05, vs. vehicle/DEX. (**d**,**e**) ^‡^P < 0.05.

To further investigate the blunted CNP-53 effect during the first week after the start of treatment, we delayed the start of treatment for one week and established the four groups (i.e., CNP/vehicle group, vehicle/vehicle group, vehicle/DEX group, and CNP/DEX group) of mice treated with the same dose of CNP-53 and/or DEX from 5 to 9 weeks of age. Growth curves are shown in Fig. 4a,b. There were no significant differences in the naso-anal length between the four groups at the start of this experiment (5 weeks old). As with the administration experiment from 4 weeks of age, naso-anal lengths were shortened in the vehicle/DEX group and restored in the CNP/DEX group (Fig. 4a). However, during the first week from the start of DEX (from 5 to 6 weeks of age), the growth velocity of the CNP/DEX group exceeded that of the vehicle/DEX group (Fig. 4b), while there was no significant difference between those in the respective two groups in the experiment from 4 weeks of age (Fig. 1d).

Each bone length of the four groups of mice at the end of the experimental period is shown in Fig. 4c. As with the result shown in Fig. 1f, skeletal impairments due to DEX were restored in the CNP/DEX group mice. The humerus, radius, ulna, femur, tibia, fibula, and lumbar vertebrae grew to a comparable length to those in the vehicle/vehicle group.

To elucidate the mechanistic difference in DEX-induced CNP-53 resistance observed in 4–5-week-old mice compared to 5–6 weeks old, we measured the expression of *Nr3c1*, which encodes the murine glucocorticoid receptor (GR) of 4-week-old mice and 5-week-old mice. The mRNA levels of lumbar vertebrae, including bone and growth plate, and costal cartilages, composed of cartilage tissue, were measured and *Nr3c1* expression was significantly reduced in the 5-week-old mice compared to the 4-week-old mice (Fig. 4d,e). Data shown in Fig. 4d,e were normalized by *Hprt* and the same result was obtained when *Ppia* was used as the reference gene; relative expression of *Nr3c1* in the lumber vertebrae normalized by *Ppia* was 1.00 ± 0.062 in 4-week-old mice and 0.70 ± 0.027 in 5-week-old mice, respectively (P < 0.05, n = 3, each) and that in the costal cartilages normalized by *Ppia* was 1.00 ± 0.053 in 4-week-old mice and 0.76 ± 0.054 in 5-week-old mice, respectively (P < 0.05, n = 3, each).

### The effect of the alteration of the start point of CNP-53 administration on GC-induced impaired skeletal growth

**Figure 5:**
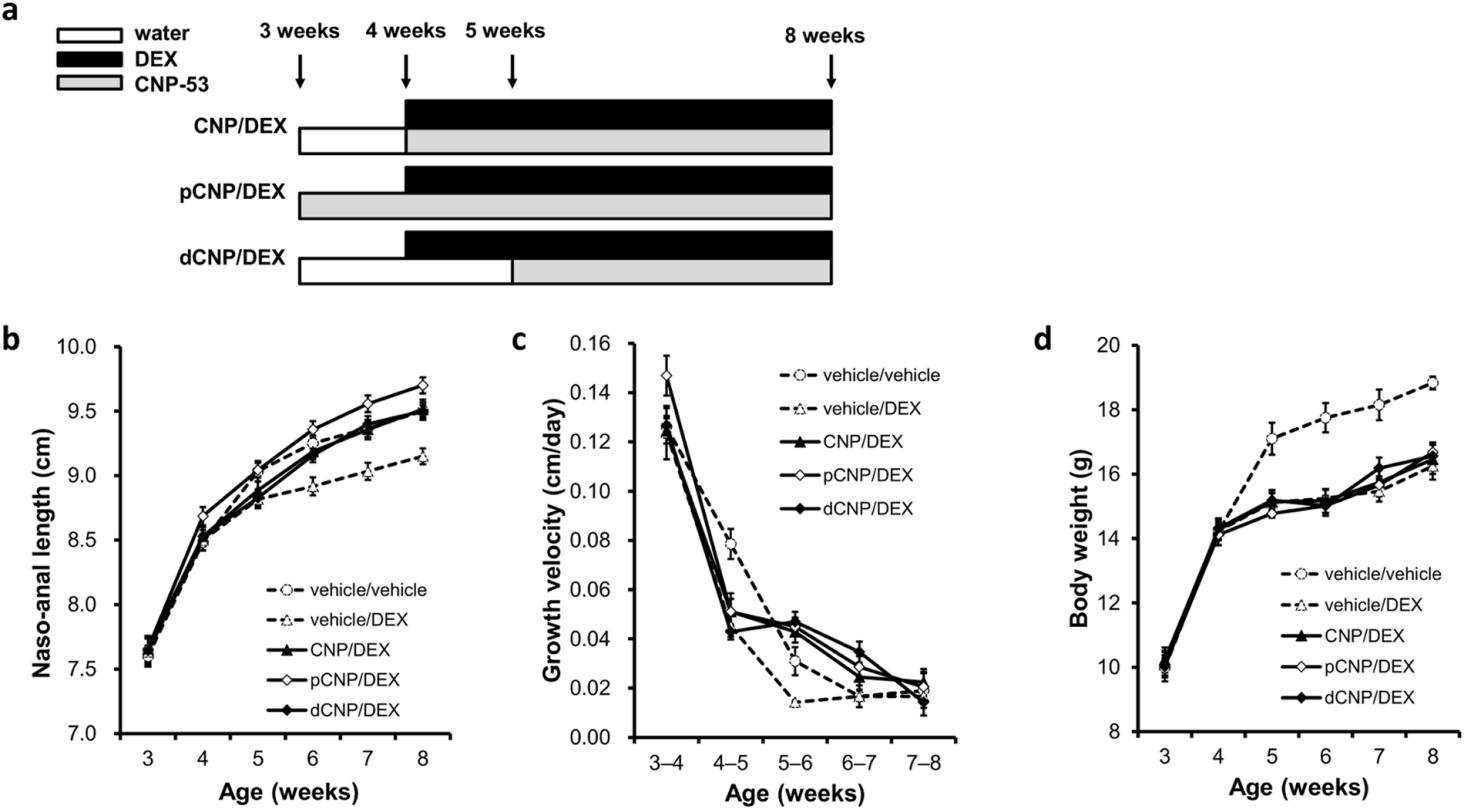
The effect of preceded or delayed CNP-53 treatment. (**a**) Schema of the time course of treatment. (**b**) Growth curves and (**c**) growth velocities of naso-anal lengths, and (**d**) body weights of CNP/DEX (▲), pCNP/DEX (◊), and dCNP/DEX (◆). Graph lines of vehicle/vehicle (○) and vehicle/DEX (Δ) depicted in Fig. 1 are also inserted into the respective graphs as dashed lines. n = 7, each, in CNP/DEX, pCNP/DEX, and dCNP/DEX.

To explore the therapeutic strategy for GC-induced growth retardation, we examined the best timing of the start of the therapy. We came up with therapeutic models mimicking GC-induced growth retardation, arranging two additional CNP/DEX groups, with preceded CNP treatment (pCNP/DEX group) and delayed CNP treatment (dCNP/DEX group). In the pCNP/DEX group, 0.5 mg/kg/day of CNP-53 was administered one week earlier. In the dCNP/DEX group, the same dose of CNP-53 was administered one week later. In both groups, 2.0 mg/kg/day of DEX was administered in the same manner as before. Schema of the time course of this experiment is depicted in Fig. 5a. As shown in Fig. 5b, in the pCNP/DEX group, the naso-anal length at 4 weeks of age was larger than that of the CNP/DEX group but from 4 weeks of age, the inclination of the growth curve was comparable to that of the CNP/DEX group. In the dCNP/DEX group, the whole growth curve overlapped that of the CNP/DEX group. Accordingly, there were no significant differences in the growth velocities among the three CNP/DEX groups (Fig. 5c). Body weight of the three CNP/DEX groups had no significant differences (Fig. 5d).

### The effects of high-dose CNP-53 administration on GC-induced impaired skeletal growth

**Figure 6:**
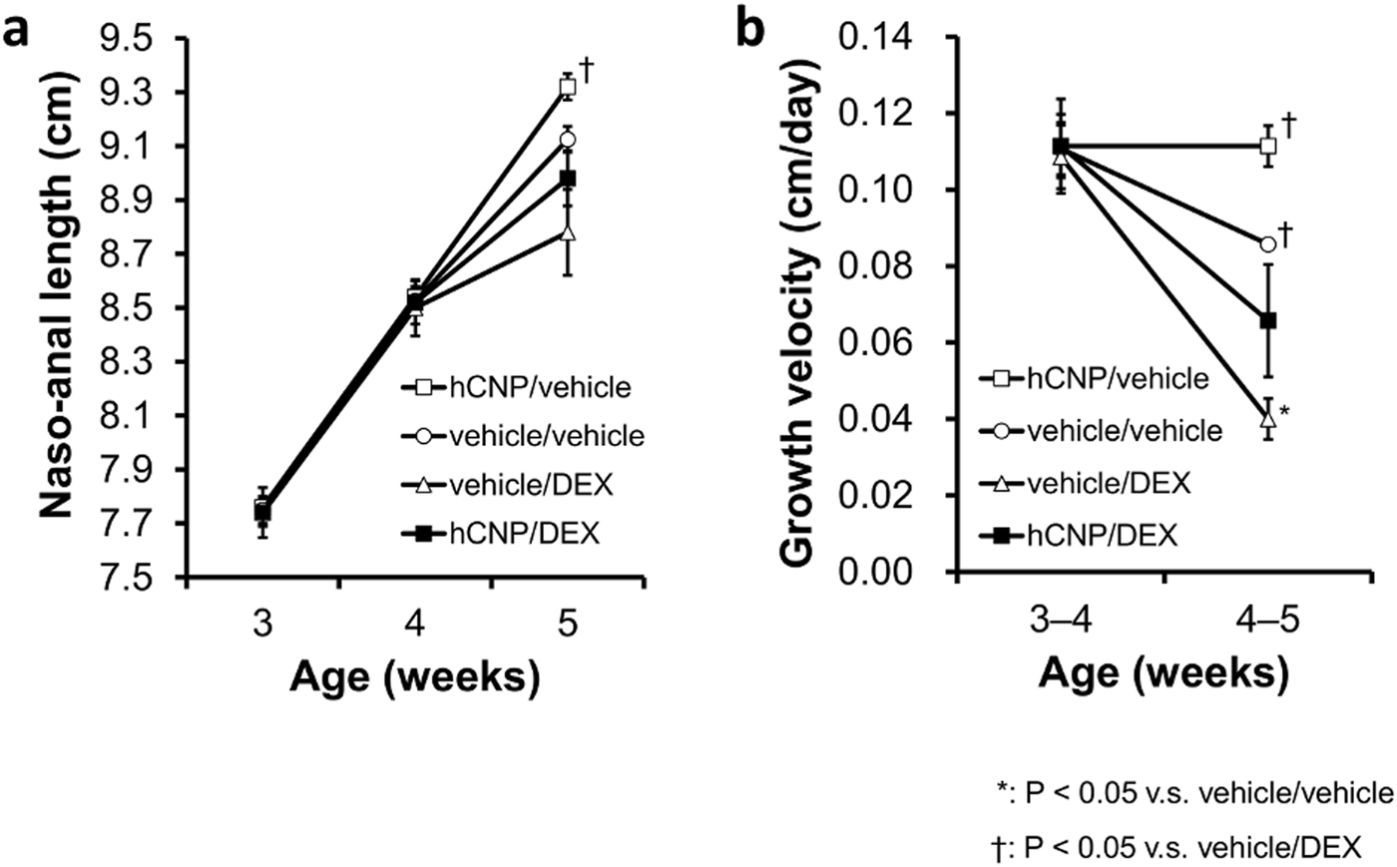
The effect of high-dose CNP-53 administration. Changes in (**a**) naso-anal length and (**b**) growth velocities of hCNP/vehicle (□), vehicle/vehicle (○), vehicle/DEX (Δ), and hCNP/DEX (■) between 4 weeks to 5 weeks of age. hCNP/vehicle, n = 4; vehicle/vehicle, n = 5; vehicle/DEX, n = 5; and hCNP/DEX, n = 5. *P < 0.05, vs. vehicle/vehicle and ^†^P < 0.05, vs. vehicle/DEX.

To further investigate the blunted CNP-53 effect during 4 to 5 weeks of age, we evaluated the efficacy of high-dose CNP-53 treatment during this period. We arranged four groups of mice, including the group treated with high-dose CNP-53: high-dose CNP/vehicle group (hCNP/vehicle group), vehicle/vehicle group, vehicle/DEX group, and high-dose CNP/DEX group (hCNP/DEX group). In each group, CNP-53, DEX, and/or vehicle were administered daily from 4 to 5 weeks of age. CNP-53 and/or DEX were administrated at the same dose, 2.0 mg/kg/day. The DEX-induced growth retardation tended to be reduced but could not be completely restored by high-dose CNP-53 during 4 to 5 weeks of age (Fig. 6a,b).

### The effect of DEX on the CNP/NPR-B system

**Figure 7:**
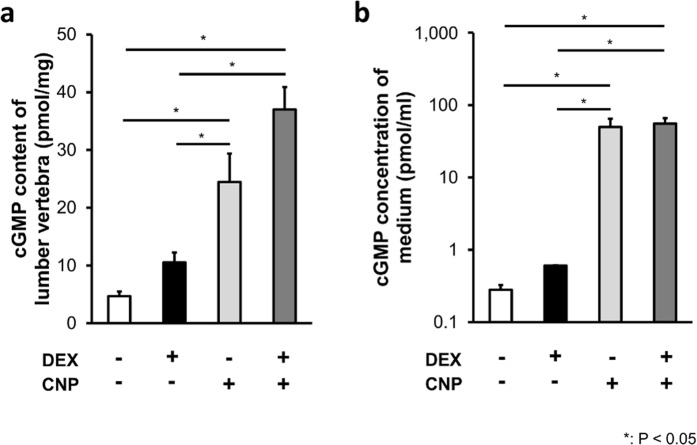
cGMP level of lumbar vertebrae and culture medium of ATDC5 cells. (**a**) cGMP content of lumbar vertebrae of mice injected with DEX, CNP-53, and/or vehicle, and (**b**) cGMP concentration of culture media of ATDC5 cells incubated with DEX, CNP-22, and/or vehicle. (**a**) n = 4 and (**b**) n = 3 each, in DEX(−)/CNP(−), DEX(+)/CNP(−), DEX(−)/CNP(+), and DEX(+)/CNP(+). *P < 0.05.

To elucidate the mechanism of DEX-induced CNP-53 resistance during 4 to 5 weeks of age, we investigated the interaction between DEX and CNP-53. We explored the effect of DEX on the CNP/NPR-B system by measuring cGMP levels at 4 weeks of age. We treated 4-week-old mice with vehicle, DEX, or 0.5 mg/kg/day of CNP-53, or 0.5 mg/kg/day of CNP-53 and DEX for 3 days and measured cGMP content in their lumber vertebrae. As shown in Fig. 7a, cGMP content was elevated in CNP-53-treated mice and DEX did not significantly change cGMP content. In addition, we also examined cGMP change in a chondrogenic cell line, in ATDC5 cells. We measured cGMP levels in the incubation medium of ATDC5 cells treated with vehicle, DEX, or CNP-22, or CNP-22 and DEX. The cGMP levels were elevated in the group treated with CNP-22, and were not altered by DEX, which was consistent with the results of our *in vivo* experiment (Fig. 7b).

### The effect of CNP and DEX on intracellular signaling in ATDC5 cells

**Figure 8:**
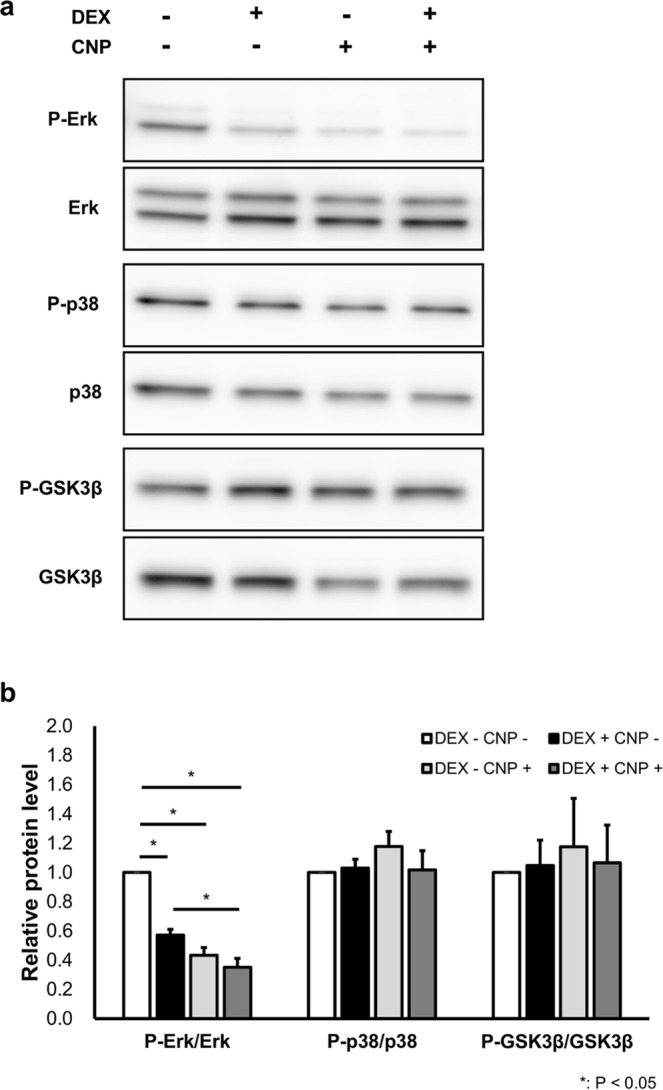
Western blotting analysis of intracellular signaling in ATDC5 cells. (**a**) Images of western blotting analysis of P-Erk, Erk, P-p38, p38, P-GSK3β, and GSK3β in ATDC5 cells cultured with DEX, CNP-22, and/or vehicle. (**b**) Signal intensities measured on images. (**b**) n = 3 each, in DEX(−)/CNP(−), DEX(+)/CNP(−), DEX(−)/CNP(+), and DEX(+)/CNP(+) in P-Erk/Erk, P-p38/p38, and P-GSK3β/GSK3β. *P < 0.05.

For further study on the interaction between DEX and CNP, we analyzed intracellular signaling of DEX and CNP in ATDC5 cells by using western blotting analysis. We extracted total protein from differentiated ATDC5 cells treated with vehicle, DEX, or CNP-22, or both CNP-22 and DEX and evaluated the phosphorylation of Erk 1/2, p38, and GSK3β. As shown in Fig. 8a,b, DEX reduced Erk 1/2 phosphorylation in ATDC5 cells. CNP-22 also reduced Erk 1/2 phosphorylation and the effect was still stronger than DEX, and CNP-22 further reduced Erk 1/2 phosphorylation even under treatment with DEX (Fig. 8a,b). As for p38 and GSK3β phosphorylation, neither DEX nor CNP-22 changed them (Fig. 8a,b). Whole gel pictures are shown in Supplementary Figure.

## Discussion

We previously reported that CNP could be an effective therapeutic agent for GC-induced growth retardation by using transgenic mice overexpressing CNP under the control of SAP promoter^35^. However, these transgenic mice had high blood CNP concentrations just after birth^36^, so they were not an appropriate model for the treatment of acquired dwarfism. Therefore, we performed an administration experiment of exogenous CNP in this study. CNP is generated by the processing of proCNP, and its active forms are CNP-22 and CNP-53. Because CNP-22 is rapidly degraded by NEP in subcutaneous tissue mainly due to its small size^38^, CNP-22 had to be administered continuously and intravenously to activate bone growth^34^. In this study, we used CNP-53 because it was a dominant form of endogenous CNP^37^ and had a higher NEP resistance than CNP-22^38^. We demonstrated that daily subcutaneous injection of CNP-53 could activate bone growth and restore GC-induced growth retardation. This is the first report that daily subcutaneous injection of CNP-53 can promote bone growth in mice, although we have recently demonstrated that continuous subcutaneous administration of CNP-53 could activate bone growth of rats^39^.

When CNP-53 and DEX were administered from 4 weeks of age, the final naso-anal length and bone length were restored in the CNP/DEX group and impaired tibial growth plates were also restored by CNP-53 (Figs 1 and 2). Nevertheless, CNP-53 failed to improve the DEX-induced growth retardation during 4–5 weeks of age. Histological analysis showed that DEX did not impair proliferative zones but impaired hypertrophic zones and that CNP did not restore hypertrophic zones during 4–5 weeks of age but restored during 5–6 weeks of age (Fig. 3). These results indicate that growth impairment due to DEX is related to the attenuation of hypertrophic zones and CNP restores the growth impairment by improving the attenuation of hypertrophic zones. This hypothesis is consistent with our past study^40^.

Because single administration of CNP-53 was effective at all ages during the experimental period, we speculated that DEX-induced growth impairment in this one week is resistant to CNP and hypothesized that the difference in age from the start of treatment resulted in CNP resistance. To verify this hypothesis, we performed an administration experiment in which the starting point of treatment was modified. When both CNP-53 and DEX were started at 5 weeks of age, CNP-53 was effective during the first week from the start of treatment (Fig. 4). When CNP-53 was started one week earlier than DEX, from 3 weeks of age, CNP-53 was not effective during 4–5 weeks of age (the first week from the start of DEX), as shown by the growth velocity depicted in Fig. 5c. Then, we treated 4-week-old mice with DEX and quadrupled the CNP-53 dose. The quadrupled dose of CNP-53 could not restore DEX-induced growth retardation during the first week with CNP resistance (Fig. 6). Furthermore, when CNP-53 was started one week later than DEX, from 5 weeks of age, avoiding the fourth week with CNP resistance, CNP-53 was effective during the first week of treatment (5–6 weeks of age) and final length was comparable to that of mice that received CNP-53 from 4 weeks of age (Fig. 5b). Taken together, there exists an age-dependent CNP-resistance when treating GC-induced impaired growth in a mouse model. This finding is very important to establish an optimal therapeutic schedule when CNP-53 is used in clinical settings.

Following this, to explore the mechanism of age-dependent CNP resistance to GC-induced impaired growth, we measured the expression of *Nr3c1* encoding GR in the lumber vertebrae and costal cartilages by using quantitative PCR technology, and found a higher expression of *Nr3c1* in the lumbar vertebrae of 4-week-old mice than that of 5-week-old mice (Fig. 4d,e). Sensitivity to GC is well studied in inflammatory diseases with GC resistance and there are several mechanisms regulating GC sensitivity such as induction of a decoy receptor^41^, suppression of histone deacetylase, and transcriptional regulation through GR^42^. Although there could be other mechanisms which explain the age-dependent GC effect, our finding that GR expression decreases in 5-week-old mice compared to 4-week-old mice is consistent with the age-dependent nature of DEX-induced CNP resistance. The result of recent clinical research exhibiting low expression of GR in children with GC-resistant nephrotic syndrome might support this notion^43^.

To further investigate the mechanism of the CNP resistance by DEX, we measured the cGMP content in lumbar vertebrae of mice treated with CNP-53 and/or DEX, expecting DEX could inhibit the CNP-53 effect at the receptor level. The results showed that DEX did not impair the CNP-53-induced elevation of cGMP content (Fig. 7a). In this case, we should consider the half-life of CNP-53 when used *in vivo*; while we could not find any reports suggesting the half-life of CNP-53, that of CNP-22 was reported to be a few minutes^44^ and therefore, the half-life of CNP-53 might not be long. There could be a diurnal variation of CNP-53 when it was injected daily and subcutaneously, and the change in naso-anal length should be dependent on the CNP-53 concentration. Although we measured the cGMP content of lumbar vertebrae 30 minutes after the last CNP-53 injection, this single measurement could be insufficient to evaluate the CNP-53 effect. To resolve this concern, we tried continuous administration of CNP-53 with an osmotic pump but failed because of uncontrollable infection in the DEX-treated group. Instead, we cultured ATDC5 cells with CNP and/or DEX as a model treated with CNP and/or DEX equably and continuously. This *in vitro* experiment also indicated DEX did not prevent CNP-53-induced cGMP elevation (Fig. 7b).

Furthermore, we investigated the alteration of intracellular signaling in chondrocytes downstream of cGMP. Although CNP/NPR-B/cGMP signaling was reported to be related to p38 MAPK^45^ and GSK3β^46^, we could not find any significant changes in the phosphorylation of p38 and GSK3β after CNP and/or DEX treatment in this study using ATDC5 cells (Fig. 8). However, when we measured Erk phosphorylation, we found that CNP, as well as DEX prevented the phosphorylation of Erk in ATDC5 cells. In addition, ATDC5 cells incubated with both CNP and DEX had lower phosphorylation levels than those with either CNP or DEX (Fig. 8). Erk MAPK signaling is reported to be inhibitory to chondrocyte differentiation and bone growth in various studies including Erk inhibition^20,47^ and overexpression^48^, and it is an established fact that suppression of Erk phosphorylation promotes endochondral bone growth. CNP prevents Erk phosphorylation as we exhibited here and had previously reported^20^, so at least part of the stimulating effect of CNP on bone growth is owed to the inhibition of Erk phosphorylation. In regard to Erk inhibition, our present result suggests that DEX has the potential to enhance growth by suppressing Erk phosphorylation. Nevertheless, it is well known that DEX induces growth retardation by induction of IGF-1 resistance^12,13^, inhibition of chondrocyte proliferation, and promotion of apoptosis of chondrocytes^10,11^. DEX-induced growth impairment is the result of these negative effects exceeding the positive effect, i.e., suppression of Erk phosphorylation. Even when Erk phosphorylation was already suppressed by DEX, a CNP effect was observed, strengthening the suppression of Erk phosphorylation (Fig. 8). In other words, CNP reinforced the latent positive effect of GC and this CNP effect did not disappear under the treatment of DEX. So CNP could overcome the inhibitory effect of DEX when the positive effect of CNP, including the suppression of Erk phosphorylation, exceeded the negative effect of DEX. Accordingly, when focusing on the growth promoting effect of Erk suppression, we can presume that when the GC effect is relatively strong, the CNP effect is masked by the Erk-related positive effect of GC, and that when the GC effect is relatively weak, CNP can restore GC-induced growth retardation by reinforcing the weak and latent positive effect of Erk suppression by GC. This speculation is consistent with the result of our present *in vivo* study, showing that CNP-53 was not effective at 4 weeks of age when the DEX effect was relatively strong, while CNP-53 was effective at 5 weeks of age when the DEX effect was relatively weak. The results of the present histological analysis exhibiting that CNP-53 was not effective in DEX-induced narrowing of the hypertrophic zone at 4 weeks of age and was effective at 5 weeks of age also support this notion. Furthermore, this result suggests that the enlargement of hypertrophic chondrocytes is very important for the endochondral bone growth promoted by CNP, which is consistent with our recent study on live imaging of cultured bone by using two-photon microscopy^40^.

In conclusion, we demonstrated the therapeutic potential of daily subcutaneous injection of exogenous CNP-53 for GC-induced growth retardation in a mouse model. We also showed the existence of CNP resistance by DEX at a specific age in this model. While clinical studies are needed, we believe that the results of this study have a great importance in determining the optimal therapy in clinical settings.

## Materials and Methods

### Animals

C57BL/6JJcl mice were purchased from Japan SLC, Inc. (Hamamatsu, Japan). All experimental procedures involving animals were approved by the Animal Research Committee, Graduate School of Medicine, Kyoto University (Permit Number: Med Kyo 17218, 18247). Care of animals and all animal experiments were conducted in accordance with the institutional guidelines of Kyoto University Graduate School of Medicine.

In our previous report using transgenic mice that had elevated circulating levels of CNP and were treated with DEX, we obtained the same qualitative results as for male and female mice on the linear growth^35^. So we chose to use female mice for the following experiments.

### Reagents

CNP-53 was purchased from PEPTIDE INSTITUTE, INC. (Ibaraki, Japan) as human CNP-53 (No. 4241-s, PEPTIDE INSTITUTE) and dissolved in water to a concentration of 50 µg/ml or 200 µg/ml. DEX was purchased from Wako Pure Chemical Industries, Ltd. (Osaka, Japan) as dexamethasone sodium phosphate (No. 040-30811, Wako), and dissolved in saline to a concentration of 200 µg/ml. CNP-53 was injected subcutaneously at a dose of 0.5 mg/kg/day or 2.0 mg/kg/day, using 50 µg/ml or 200 µg/ml of CNP-53 solution, respectively. The dose of 0.5 mg/kg/day was determined in accordance with our previous report in which the dose of CNP-53 restored the dwarfism of CNP knockout rats^39^. The dose of 2.0 mg/kg/day was determined as a high dose of therapeutic CNP-53. DEX was injected subcutaneously at a dose of 2.0 mg/kg/day. The dose was determined in accordance with our previous report in which the dose of DEX caused significant growth retardation^35^. As vehicles, water and/or saline were injected at a dose of 10 ml/kg/day. The volume of the vehicle was equivalent to that of the CNP-53 or DEX solution.

### Administration of reagents to mice

DEX and CNP-53 were injected from 4 to 8 weeks or from 5 to 9 weeks of age. The dose of DEX was 2.0 mg/kg/day and that of CNP-53 was 0.5 mg/kg/day. Four groups of mice (CNP/vehicle, vehicle/vehicle, vehicle/DEX, and CNP/DEX groups) were arranged in each experiment. The numbers of mice used were 7, 6, 6, and 6 (4–8 weeks of age), and 5, 4, 4, and 4 (5–9 weeks of age), respectively.

In delayed and preceded CNP-53 experiments, the start points of CNP-53 were altered: delayed CNP-53 was injected from 5 to 8 weeks of age and preceded CNP-53 was injected 3 to 8 weeks of age. DEX was injected from 4 to 8 weeks of age. The dose of DEX was 2.0 mg/kg/day and that of CNP-53 was 0.5 mg/kg/day. In this series of experiments, three groups of mice (dCNP/vehicle, pCNP/DEX, and CNP/DEX groups) were examined. The number of mice was 7 in each group.

In high-dose CNP-53 experiment, DEX and CNP-53 were injected from 4 to 5 weeks of age. The dose of DEX was 2.0 mg/kg/day and that of CNP-53 was 2.0 mg/kg/day. Four groups of mice (hCNP/vehicle, vehicle/vehicle, vehicle/DEX, and hCNP/DEX groups) were arranged in this experiment, and the numbers of mice used were 4, 5, 5, and 5, respectively.

### Evaluation of the growth of mice

The vehicle, DEX, or CNP-53, or CNP-53 and DEX were injected subcutaneously every day. The naso-anal length and body weight were measured weekly under isoflurane-induced anesthesia. During the measurement of the naso-anal length, the cranium of the target mouse was fixed and the body was stretched to its fullest extent. The validity of this method was discussed in our past study^35^.

### Skeletal analysis

Mice were treated with vehicle, DEX, or CNP-53, or CNP-53 and DEX for 4 weeks. After the treatment, the length and width of the cranial bone and the lengths of the humerus, radius, ulna, femur, tibia, fibula, and lumbar vertebrae of each mouse were measured on soft X-ray film. Lengths of the humerus, radius, ulna, femur, tibia, and fibula were averages of the right and left sides. We measured the span from the first lumbar vertebra to the fifth as the length of the lumbar vertebrae.

### Histological analysis

Mice were sacrificed after the treatment with vehicle, DEX, or CNP-53, or CNP-53 and DEX, and their tibias were collected. These tibias were fixed in 10% formalin neutral buffer solution and decalcified with 10% EDTA for 2 weeks before they were embedded in paraffin and cut longitudinally to analyze the growth plates. After the bone sections were deparaffinized and rehydrated, alcian-blue staining of tibial growth plates was performed using Alcian Blue Stain Solution (No. 37154-15, Nacalai, Kyoto, Japan). The widths of the whole growth plate, proliferative zone, and hypertrophic zone were measured on three intermittent sections per each specimen: chondrocytes flattened and arranged into columns were defined as proliferative chondrocytes and swollen chondrocytes were defined as hypertrophic chondrocytes. The widths were measured at three randomly chosen points for each section and the average of three measured values was regarded as the width of the section. The average of three widths of the sections of each specimen was regarded as the width of the whole growth plate, or its proliferative or hypertrophic zone.

### Quantitative RT-PCR analysis

Four and 5-week-old mice were sacrificed and resected their lumbar vertebrae and costal cartilages, removing muscle tissue and nerve tissue carefully. Total RNA was extracted from their lumbar vertebrae and costal cartilages using RNeasy Lipid Tissue Mini Kit (No. 74084, QIAGEN). 1 µg of total RNA was reverse-transcribed using ReverTra Ace (No. TRT‐101, TOYOBO Life Science, Osaka, Japan). Quantitative PCR analysis was performed using THUNDERBIRD SYBR qPCR MIX (No. QPS-201, TOYOBO Life Science) with the StepOnePlus™ Real-time PCR System (Thermo Fisher Scientific, Massachusetts, USA). Results were normalized using hypoxanthine guanine phosphoribosyl transferase (Hprt) and peptidylprolyl isomerase A (Ppia) as reference genes, which are reported to be the most suitable genes for normalization in quantitative PCR assay in chondrocytes^49^. The primers used in this analysis were as follows: Nr3c1, forward AAAGAGCTAGGAAAAGCCATTGTC and reverse TCAGCTAACATCTCTGGGAATTCA; Hprt, forward GGACCTCTCGAAGTGTTGGATAC and reverse GCTCATCTTAGGCTTTGTATTTGGCT; Ppia, forward CGCGTCTCCTTCGAGCTGTTTG and reverse TGTAAAGTCACCACCCTGGCACAT.

### Cell culture

A chondrogenic cell line, ATDC5 cells were purchased from RIKEN CELL BANK (No. RCB0565, RIKEN CELL BANK, Tsukuba, Japan). The cell line was authenticated by RIKEN CELL BANK. were maintained with Dulbecco’s modified Eagle’s Medium/Nutrient Mixture F-12 Ham (No. D6421, SIGMA) containing 5% fetal bovine serum (No. 10270-106, Thermo Fisher Scientific), 100 U/ml penicillin, and 100 μg/ml streptomycin (No. 26253-84, Nacalai) at 37 °C in a humidified atmosphere of 5% CO_2_. The medium was replaced every other day.

### Measurement of cGMP levels

Four-week-old mice were treated with vehicle, DEX, or CNP-53, or CNP-53 and DEX for 3 days. These mice were sacrificed 30 minutes after the last injection and their lumber vertebrae were resected. Samples were homogenized in 5% trichloroacetic acid (TCA) and TCA was extracted into water-saturated ether. The obtained cGMP solutions were acetylated to increase the sensitivity of the following assay. cGMP levels of the lumbar vertebrae were measured by cGMP ELISA kit (No. 581021, Cayman Chemical, Michigan, USA).

For *in vitro* studies, ATDC5 cells were plated at 1.0 × 10^5^ cells/well in 6-well tissue culture plates and cultured with 10 µg/ml bovine insulin (No. 10516, SIGMA) for 14 days to differentiate into proliferative chondrocytes which are abundant in NPR-B^50^. After differentiation, ATDC5 cells were incubated with vehicle or 10^−7^ M DEX for 30 minutes, and then vehicle or 10^−7^ M CNP-22 (No. 4229-v, PEPTIDE INSTITUTE) were added into the medium for 24 hours. Culture medium was acetylated to increase the sensitivity of the following assay and cGMP levels were measured directly by cGMP ELISA kit.

### Western blotting analysis

ATDC5 cells were plated in 6-well tissue culture plates and differentiated for 14 days as described above. Differentiated ATDC5 cells were incubated in insulin-free medium for 2 days. Then after the incubation with vehicle or 10^−6^ M DEX^51^ for 30 minutes, vehicle or 10^−6^ M CNP-22^20^ was added into the medium for 30 minutes. Total protein was extracted from ATDC5 cells by RIPA buffer containing SDS solution and a protease inhibitor cocktail (No. 08714-04, Nacalai), which was supplemented with phosphatase inhibitors (No. 07574-61, Nacalai). Western blotting was performed using the following primary antibodies: Erk 1/2 (No. 4695S, Cell Signaling Technology), Phospho-Erk 1/2 (No. 4376S, Cell Signaling Technology), p38 (No. 9212S, Cell Signaling Technology), Phospho-p38 (No. 9211S, Cell Signaling Technology; RRID), GSK3β (No.9315S, Cell Signaling Technology), and Phospho-GSK3β (No. 9323 S, Cell Signaling Technology).

### Statistical analysis

Data are expressed as means ± SE. Statistical analysis of the data was performed using either Student’s t test or one-way factorial analysis of variance (ANOVA), followed by the Tukey-Kramer test as a post hoc test. The differences were considered significant when P values were less than 0.05.

## Supplementary information

Dataset 1

## Data Availability

All data generated or analyzed during this study are included in this article.
