# Supplementary material for: Exogenous C-type natriuretic peptide therapy for impaired skeletal growth in a murine model of glucocorticoid treatment
Source: Sci Rep. 2019 Jun 12;9:8547. doi: 10.1038/s41598-019-44975-w (PMC6561908; doi:10.1038/s41598-019-44975-w)
Supplement: Supplementary file 1 — Dataset 1 [file 41598_2019_44975_MOESM1_ESM.docx]

**Exogenous C-type natriuretic peptide therapy for impaired skeletal growth in a murine model of glucocorticoid treatment**

**Yohei Ueda^1^, Akihiro Yasoda^1*^, Keisho Hirota^1^, Ichiro Yamauchi^1^, Takafumi Yamashita^1^, Yugo Kanai^2^, Yoriko Sakane^3^, Toshihito Fujii^1^ and Nobuya Inagaki^1^**

^1^Department of Diabetes, Endocrinology and Nutrition, Kyoto University Graduate School of Medicine, 54 Shogoin-Kawahara-cho, Sakyo-ku, 606-8507 Kyoto, Japan
^2^Department of Diabetes and Endocrinology, Osaka Red Cross Hospital, 5-30 Fudegasaki-cho, Tennoji-ku, 543-8555 Osaka, Japan

^3^Preemptive Medicine and Lifestyle Related Disease Research Center, Kyoto University Hospital, 54 Shogoin-Kawahara-cho, Sakyo-ku, 606-8507 Kyoto, Japan

*****[ayasoda@kuhp.kyoto-u.ac.jp](mailto:ayasoda@kuhp.kyoto-u.ac.jp)

**Phopho-Erk**


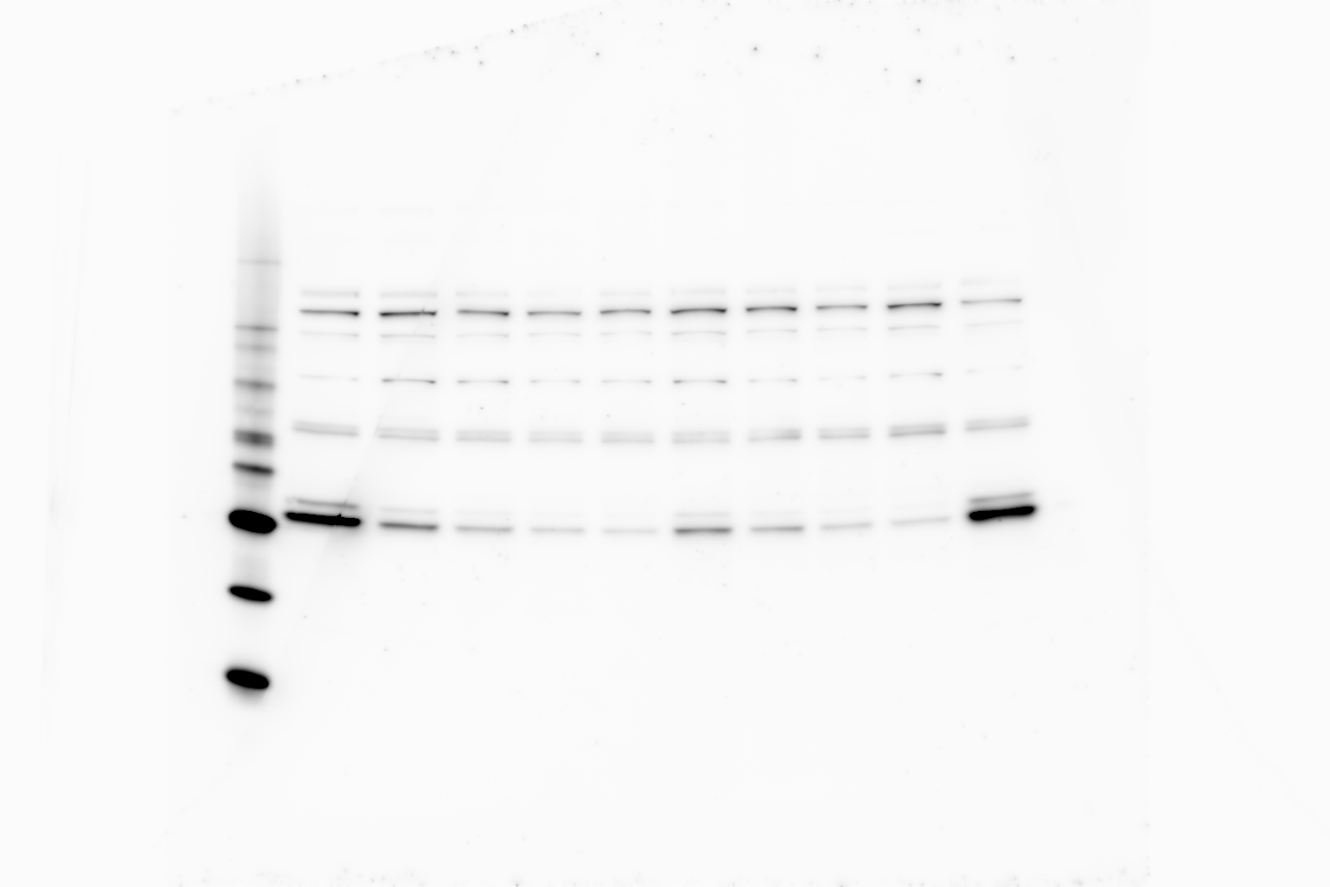


CNP - - + +

DEX - + - +

20k

30k

40k

50k

60k

42kDa

44kDa

**Erk**


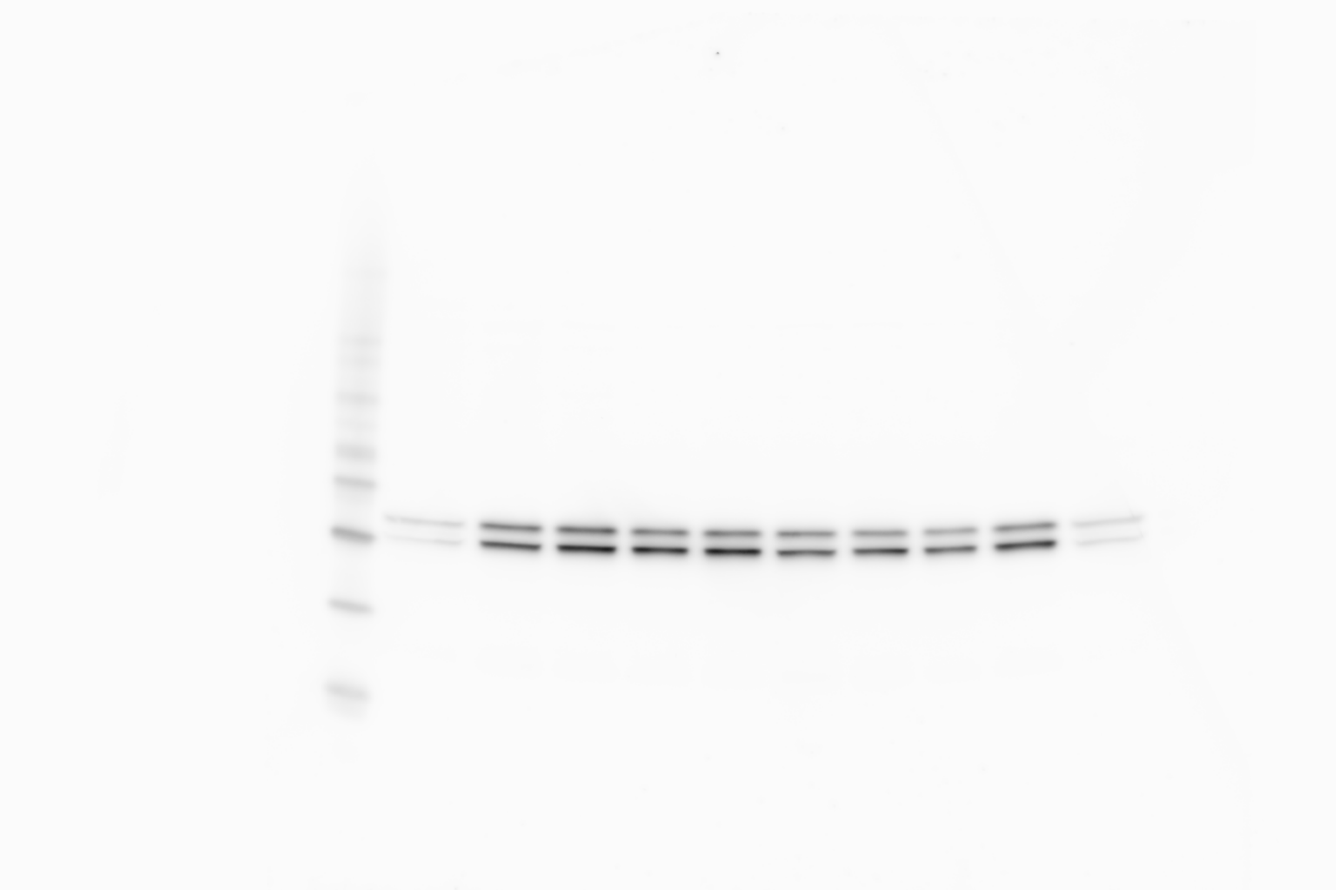


CNP - - + +

DEX - + - +

20k

30k

40k

50k

60k

42kDa

44kDa

**Phospho-p38**


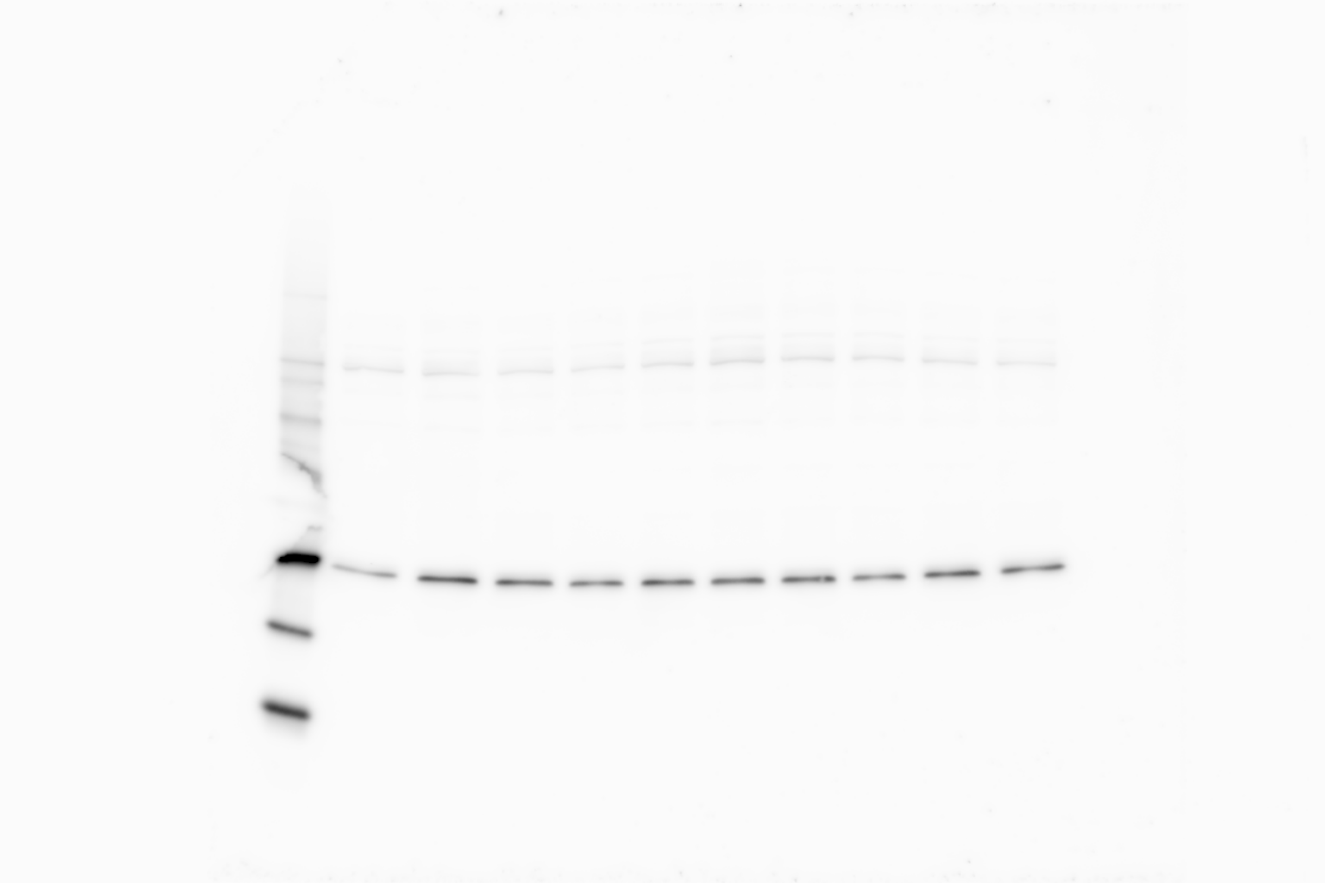


CNP - - + +

DEX - + - +

20k

30k

40k

50k

43kDa

**p38**


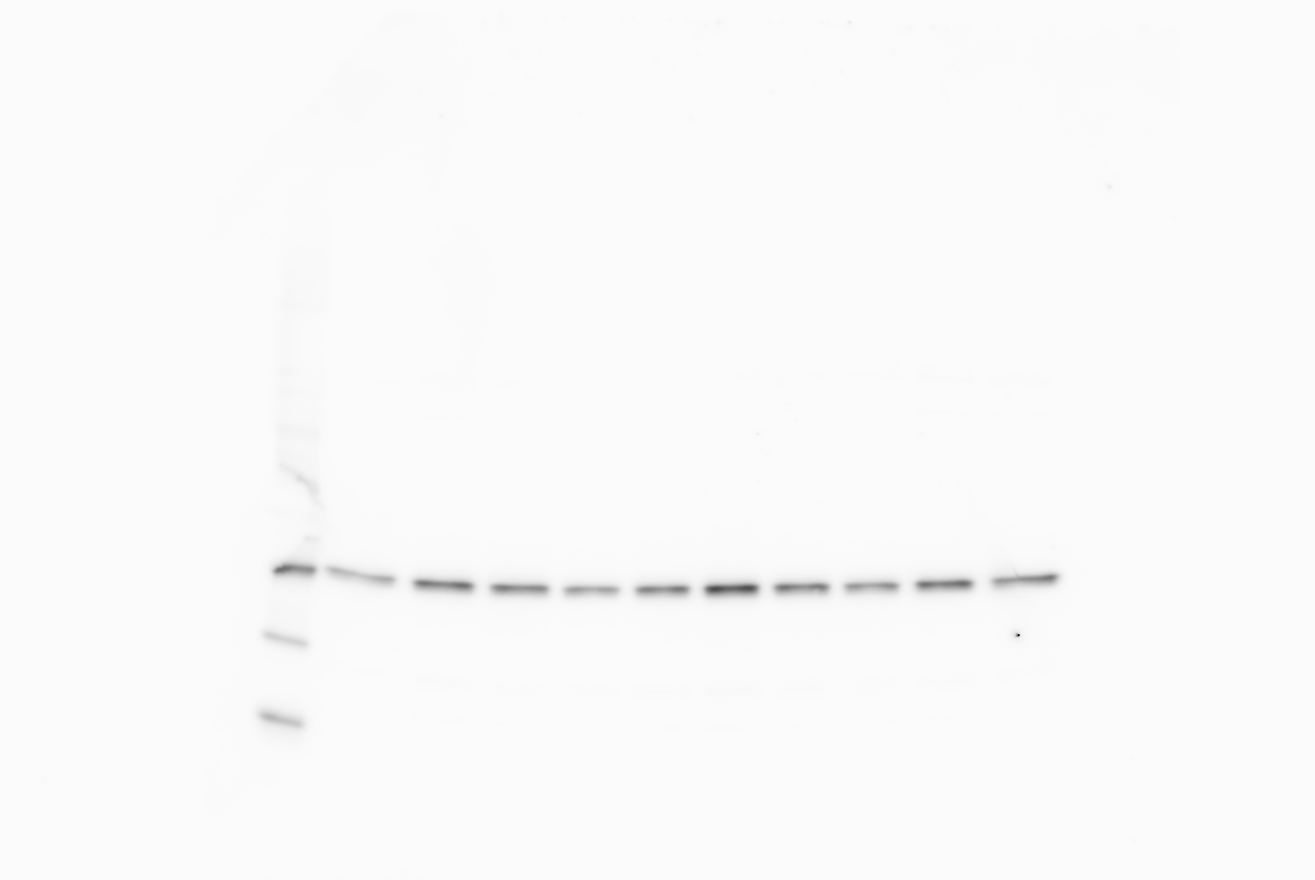


CNP - - + +

DEX - + - +

40kDa

20k

30k

40k

50k

**Phopho-GSK3β**


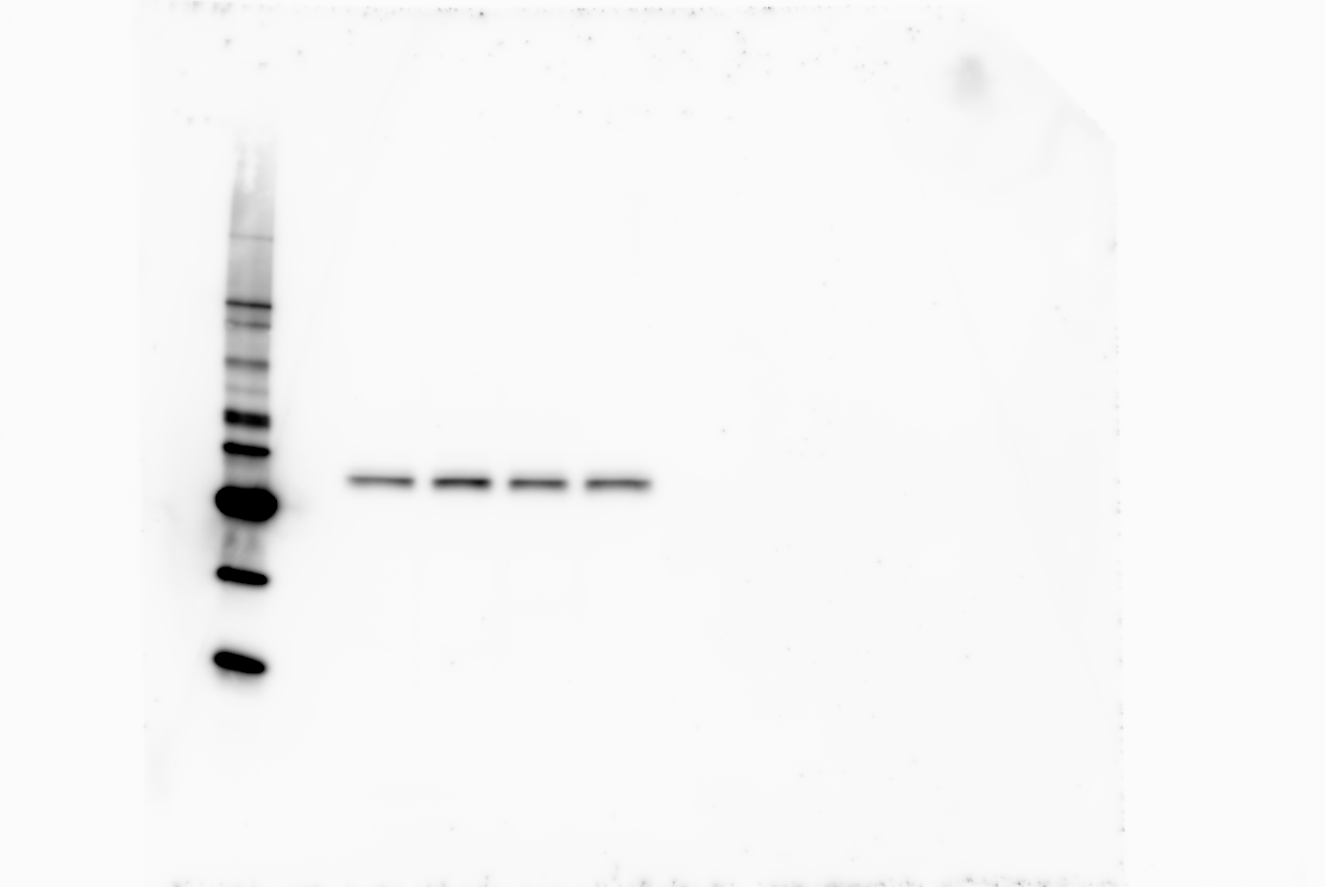


46kDa

- - + +　CNP

- + - +　DEX

20k

30k

40k

50k

60k

**GSK3β**


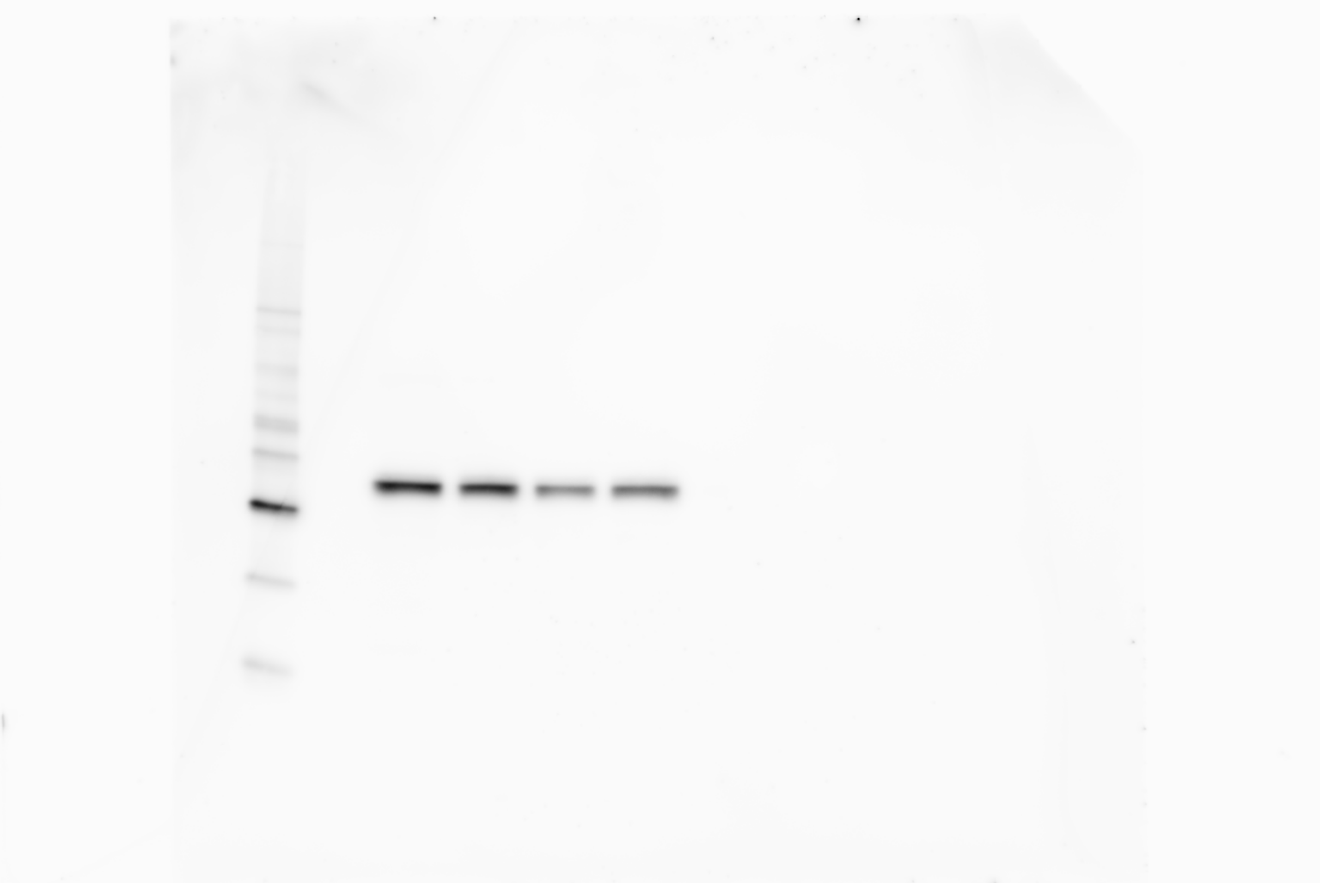


20k

30k

40k

50k

60k

46kDa

- - + +　CNP

- + - +　DEX

**Supplementary figure.** Whole gel pictures of western blotting analysis in Figure 8. Presence or absence of DEX and CNP is specified under the corresponding areas cropped for Figure 8.
